# Visuo-dynamic self-modelling of soft robotic systems

**DOI:** 10.3389/frobt.2024.1403733

**Published:** 2024-06-05

**Authors:** Richard Marques Monteiro, Jialei Shi, Helge Wurdemann, Fumiya Iida, Thomas George Thuruthel

**Affiliations:** ^1^ Bio-Inspired Robotics Lab, University of Cambridge, Cambridge, United Kingdom; ^2^ Department of Mechanical Engineering, University College London, London, United Kingdom; ^3^ Department of Computer Science, University College London, London, United Kingdom

**Keywords:** soft robotics, modelling and control, machine learning, recurrent neural net (RNN), optimal control

## Abstract

Soft robots exhibit complex nonlinear dynamics with large degrees of freedom, making their modelling and control challenging. Typically, reduced-order models in time or space are used in addressing these challenges, but the resulting simplification limits soft robot control accuracy and restricts their range of motion. In this work, we introduce an end-to-end learning-based approach for fully dynamic modelling of any general robotic system that does not rely on predefined structures, learning dynamic models of the robot directly in the visual space. The generated models possess identical dimensionality to the observation space, resulting in models whose complexity is determined by the sensory system without explicitly decomposing the problem. To validate the effectiveness of our proposed method, we apply it to a fully soft robotic manipulator, and we demonstrate its applicability in controller development through an open-loop optimization-based controller. We achieve a wide range of dynamic control tasks including shape control, trajectory tracking and obstacle avoidance using a model derived from just 90 min of real-world data. Our work thus far provides the most comprehensive strategy for controlling a general soft robotic system, without constraints on the shape, properties, or dimensionality of the system.

## Introduction

Building computational models that prescribe the relation between actuator input and robot motion is vital for robot control. These computational models can range from geometric kinematic models to fully dynamic ones. Soft robotic devices pose an imposing modelling challenge due to their nonlinear dynamics, high degrees of freedom and time-variant material properties (Iida and Laschi, 2011; Rus and Tolley, 2015; George Thuruthel et al., 2018a; Della Santina et al., 2023). Currently, reduced-order models, whether derived analytically or constructed using data-driven approaches, are employed to model and control these systems (George Thuruthel et al., 2018a; Tabak, 2019; Della Santina et al., 2023). Given the variations in their design and actuation methods, a universal modelling framework for these systems, however, has yet to emerge.

A common strategy for the modelling and control of soft robots is to restrict their motions to a quasi-static regime. This permits the development of kinematic models centered around their stable states, greatly simplifying the modelling challenge. The Constant Curvature (CC) model is the one such commonly derived kinematic model for cylindrical soft robots, where each section of a soft robot can be represented by the arm length, curvature and its angle (Camarillo et al., 2008; Webster and Jones, 2010; Mutlu et al., 2014). Higher dimensional models with increased accuracy have also been proposed. These include the variable Constant Curvature (Mahl et al., 2013; Mahl et al., 2014) (VCC), the Piece-wise Constant Curvature (PCC) (Hannan and Walker, 2003; Li et al., 2018), the Spring-Mass-Damper model (Zheng, 2012), the Cosserat Rod (Renda et al., 2012; Renda et al., 2014), beam-theory models (Camarillo et al., 2009) and Finite Element models (FEM) (Duriez, 2013; Goury and Duriez, 2018). Some of these models can be extended to incorporate dynamic properties. For instance, fully dynamic models have been developed using the CC assumption in several works (Kapadia and Walker, 2011; Falkenhahn et al., 2014; Kapadia et al., 2014; Falkenhahn et al., 2015). Likewise, kinematic models grounded in PCC principles have found applications in tasks such as impedance control during interactions with unstructured environments (Della Santina et al., 2018). They have also been employed in scenarios involving Model Predictive Control (Spinelli and Katzschmann, 2022), while Cosserat Rod models have been utilized for sliding mode control (Alqumsan et al., 2019).

Analytical models of these soft robots are constructed using simplified assumptions about their deformation, which can lead to disparities from real-world scenarios, especially when the soft robot’s structure and design change. To tackle this limitation, learning-based methods offer a solution by directly training models specific to each individual robot system. Several static controllers that directly learn mappings from the task space coordinates to the actuator space have been proposed, with different strategies for learning the ill-defined inverse mapping (Rolf, 2012; Giorelli et al., 2013; Giorelli et al., 2015; George et al., 2017). Similarly, task-space dynamic models can also be directly learned for open-loop control (Thuruthel et al., 2017; Satheeshbabu et al., 2019) or closed-loop dynamic control (George Thuruthel et al., 2018b; Gillespie et al., 2018; Haggerty et al., 2023). Regardless of whether the approach is analytical or learning-based, and whether it focuses on static or dynamic modeling, all these techniques significantly reduce the complexity of the state-space to make modeling feasible. While such models excel in task-space control, especially with feedback, they are not sufficient for more general control tasks.

**FIGURE 1:**
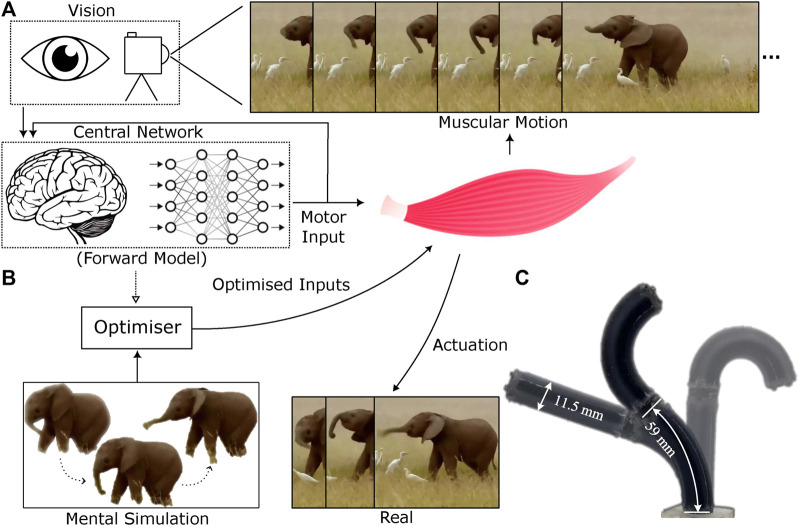
Visuo-dynamic self-modelling and control pipeline **(A)**. The system first learns a forward dynamics model in the visual space using efferent action signals and afferent visual feedback **(B)**. The forward model is then used to plan control sequences within an optimization process **(C)**. The soft robotic manipulator, STIFF-FLOP, used for experimental validation. Elephant image obtained from URL https://timesofindia.indiatimes.com/videos/amazing-but-true/this-baby-elephant-loves-to-play-with-its-trunk/videoshow/88415981.cms

The central concept of this research is to acquire dynamic sensorimotor models directly within the visual domain through a self-supervised process (as illustrated in Figure 1). This enables us to develop a task-agnostic dynamic model without any prior knowledge or assumptions about the robot morphology and dynamics. This visual simulator bears similarities to the notion of a body schema in cognitive sciences (Figure 1A), though it lacks the multimodal aspects necessary for self-recognition, as discussed in prior research (Rochat, 1998; Sturm et al., 2009; Hoffmann et al., 2010). In the field of robotics, there has been a growing interest in data-driven self-modeling techniques, ranging from kinematic modeling, exemplified by joint configuration estimation for in-hand manipulation (Hang et al., 2021), to more intricate 3-D full-body models (Chen et al., 2022). There is also a great depth of study in imitation learning or behaviour cloning, that avoids the need of an explicit model (Zhang, 2018; Ito et al., 2022; Wang et al., 2022; Chi, 2023). In our earlier work, we demonstrated the development of static shape controllers for soft robots using a self-modeling approach (Almanzor et al., 2023). This study introduces a learning-based method to comprehensively model the full dynamics of general robotic systems, eliminating the need to confine the robot’s state-space within a small finite region. No prior knowledge about the robot structure, dynamics, or dimensionality of the state-space is required, making the approach highly generalizable to any robotic system that is observable and acts on a fixed background setup.

Given the observed visual space of a generic soft robotic system $\Psi \in R^{D}$ , the unknown state-space of the soft robot $x\in R^{M}$ and the control input $u\in R^{N}$ , the forward dynamics can bewritten as:

$$\begin{array}{c} \dot{x}=f\left(x,u\right)+\eta_{i}\\ \Psi =h\left(x,u\right)+\eta_{o} \end{array}$$

Where both *f* and *h* are modeled non-linear functions and *η* represents unmodeled noise.

For a general soft robotic system, the state-space dimensions are much larger than the input space and is unknown (*M* > *N*). Full observability is challenging to be guaranteed using a data-driven method; however, as the dimensionality of the observation space increases the more likely we obtain full observability (as *D*/*M* ≫ 1). In that case, the visuo-dynamic mapping can be obtained in the discrete form shown below:

$$\begin{array}{c} x_{i+1}=f\left(x_{i},u_{i}\right)\\ \Psi_{i+1}=h\left(x_{i}\right) \end{array}$$

Where the subscripts *i*, *i* + 1 represent the variables evaluated at these respective discrete time intervals. In the above, we have chosen f and h such that the output function h is only a function of the state-space variable.

**FIGURE 2:**
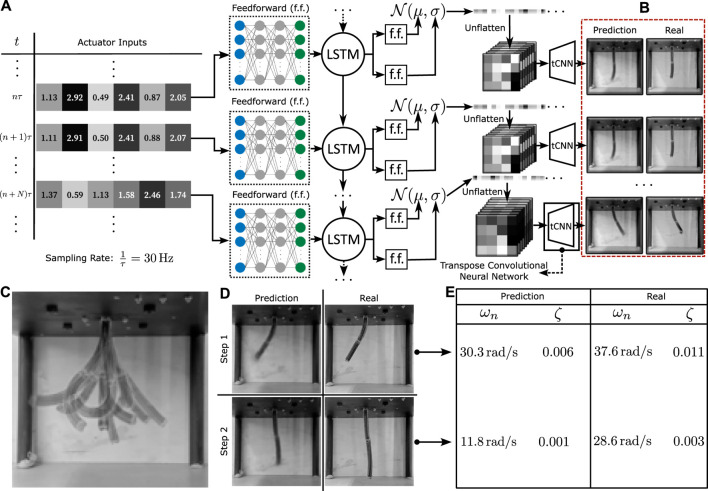
Network architecture and learning performance **(A)**. The neural network architecture that maps from actuator inputs to output image predictions. The low dimensional actuator data is passed through a time-dependent LSTM RNN network before being placed through a probabilistic generative model and a transpose CNN upscaler that transforms the data into the required high dimensional spatio-temporal image sequences. **(B)** Qualitative comparison of the predicted and training data. **(C)** Overlayed visualisation of the robot’s motion capabilities. **(D)** Comparison of predicted and real data for two step-inputs. **(E)** Relative stiffness and damping factor estimates for the two-step inputs.

The shown visuo-dynamic mapping is a transformation from a low dimensional input space [*N* = 6, for the experimented STIFF-FLOP manipulator (Fraś, 2015)] to a high-dimensional output space (*D* = 128 × 128), with recursive feedback. We hypothesize that this mapping can be learned efficiently with a long short-term memory (LSTM) Generative Decoder architecture (Figure 2A). long short-term memory networks (LSTMs) are a type of gated Recurrent Neural Networks (RNNs), which conditions the value of weights on the context via the use of gates and a memory state (Goodfellow et al., 2016). These transform the efferent control signals ${\left\{u_{i}\right\}}_{i=0}^{T}$ to a time dependent latent space, here modeled to be ${\left\{x_{i}\right\}}_{i=0}^{T}$ , effectively approximating f as a function $\hat{f}$ . This dynamic system approximation is guaranteed to be feasible for RNN models, as confirmed by Schäfer et al. (2006) Upscaling of this latent space to the shape variables ${\left\{\Psi_{i}\right\}}_{i=0}^{T}$ is done using a probabilistic generative model (Ng and Jordan, 2001), such as a variational autoencoders (VAEs) (Kingma and Welling, 2013), together with a transpose convolutional neural network (tCNN) decoder (Dumoulin and Visin, 2018), which together attempt to approximate g as $\hat{g}$ . VAEs have a continuous and smooth latent space, making them well suited for the given task and has better generalization capabilities (Kingma and Welling, 2013). It is also hoped that the Gaussian generative model is capable of modelling small white noise observed in the training data, and provide a smoother average for the model functions. Once the visuo-dynamic model is learned, any optimization-based controller can be employed to generate the control policy. The reference trajectories can be provided in the visual space directly or transformed to the visual space using camera calibration data.

**FIGURE 3:**
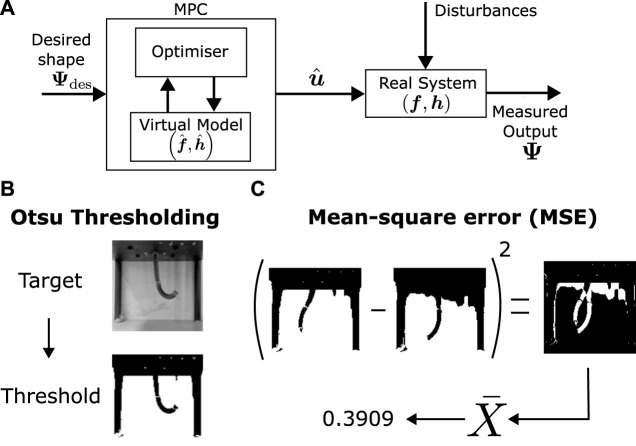
Control architecture and error calculation **(A)**. Optimization based control architecture. **(B)** Otsu thresholding (Otsu, 1979) for minimising noise and brightness. **(C)** Mean-squared error calculation from two images.

An open-loop MPC controller controller architecture is used–effectively performing simple trajectory optimisation (see Figures 1B, 3). This minimises the error between a set of target shapes ${\left\{\Psi_{\text{des},i}\right\}}_{i=0}^{T}$ and predicted shapes with the model ${\left\{{\hat{\Psi }}_{i}\right\}}_{i=0}^{T}$ by varying the control input ${\left\{u_{i}\right\}}_{i=0}^{T}$ . A dual annealing global optimiser (Xiang et al., 1997) is used for optimisation purposes, and the optimised control inputs are sent to the real robot. As an open loop method, this approach is prone to disturbance errors, previously written as the unmodeled noise *η*_*i*_ and *η*_*o*_. Through a controlled experimental setup, disturbances can be minimised therefore allowing for the validity of the proposed method.

In this work, we present a comprehensive experimental analysis of the learned visuo-dynamic model and the controller using the STIFF-FLOP manipulator. Learning performance is assessed through metrics like mean-squared loss, visual alignment of predicted and actual shapes, and oscillatory behaviour analysis. Control performance is evaluated across objectives including static and dynamic shape control, constrained manipulation, and obstacle avoidance. The system’s adaptability is verified via generalizability tests involving hand-drawn shape matching and precise tip tracking. The results underscore the approach’s robustness in tackling complex control scenarios, emphasizing its wide-ranging applicability.

## Materials and methods

### Stiff-flop design

The soft robot employed in this study is the STIFF-FLOP robotic manipulator (Fraś, 2015; Abidi, 2018), a versatile design inspired by biomimicry, particularly drawing inspiration from structures observed in the elephant trunk and the octopus arm. These inspirations were specifically chosen for their inherent adaptability, flexibility, and agility to engage with unstructured environments. The primary intended application of the STIFF-FLOP is in minimally invasive surgery, where intrinsic safety and dexterity, including elongation, omni-directional bending, and stiffness variation, are of paramount importance.

In this work, a miniaturised version of the STIFF-FLOP arm has been developed, which consists of two 59 mm robotic segments. The total diameter of the robot’s arm is 11.5 mm, maintaining a central cavity to permit the completion of surgical tasks. The fabrication details of this robot are reported in Almanzor et al., 2023. The two-segment STIFF-FLOP robotic manipulator is pneumatically driven via six 1 mm silicone pipes. To elaborate, each robotic segment has six actuation chambers, while two adjacent chambers are connected as one pair. The pressure in each of these chamber pairs is individually controlled, offering a high level of motion versatility and safe interaction capabilities, as demonstrated in Figure 1C. The maximum operating gauge pressure of the robot is set as 150 kPa.

### Robot setup

The STIFF-FLOP is configured to hang upside-down from a wooden platform. It is pneumatically powered with the help of six *SMC 6L/min* pneumatic regulators, each capable of operating at a maximum output of 500 kPa. These regulators are connected to a constant 150 kPa gauge pressure source, which ensures that the entire system operates within the safe operating range of the STIFF-FLOP, thereby avoiding any undue risk to the robot’s structural integrity. The dimensions of the robot platform are 20 × 20 cm, with the robot placed in the middle. These dimensions allow the robot to hang freely from the top and traverse the whole environment constrained only by the maximum input pressures. Figure 2C visually demonstrates the range of motion of the robot by superposing various poses.

The pneumatic regulators are powered by a *Rapid SPS-9602* power supply delivering 24 V to the connected network. The regulators feature an input range from 0 to 10 V and are linked to a *MC Measurement Computing USB-3103* digital-to-analogue converter (DAC) device. This device receives digital voltage values from a MATLAB code executed on a nearby laptop, offering direct control over the manipulator’s operation. The MATLAB code imposes a 3 V maximum limit on the applied input, corresponding to a 50 kPa pressure limit from the system.

For the purposes of data collection and subsequent deep visual analysis, a *Logitech BRIO Webcam* is used. This camera is capable of capturing 4K resolution images and is equipped with an autofocus property. The camera is secured in a fixed position using clamps to ensure that the data gathered retains the same perspective and orientation throughout the experiment. Although no depth layer is measured, the model still gains some 3D information from small brightness variations as well as the STIFF-FLOP’s texturing and colour scheme. For instance, identifying the white colored tip of the robot (in contrast to its black body) can easily determine whether it is pointing forwards or backwards.

The communication between the MATLAB program and the STIFF-FLOP is done via MATLAB, sending six voltage signals within the range of 0–3 V to the robot, simultaneously recording each signal transmitted. The camera captures the manipulator’s motion in response to the given voltage signals.

The duration of this communication loop—transmitting voltage inputs to the robot and receiving image output—sets the sampling rate for the data. Although this rate can fluctuate due to noise and variances in the I/O transfer protocols, it averages around 30 Hz, meaning each loop takes approximately 33.3 ms to complete. This communication speed sets the limit for how rapidly input can be altered within the robot, and subsequently, a limit on the maximum speed of the dynamics.

To obtain training and validation data, a motor babbling algorithm is employed. This algorithm randomly selects a new input as well as the time to reach this new input, which is varied between 1 and 5 s, well above the sampling rate of the communication path. Given enough such samples, the collected data can comprise a broad range of dynamic motions with clear temporal dependencies while avoiding very fast chaotic motion that would result from changes in lower time scales.

Post-data collection, the recorded inputs and corresponding outputs are resampled to the average frequency of 30 Hz using linear interpolation, justified by the rapid average communication loop speed. Additionally, the images are processed by cropping to focus on the STIFF-FLOP, converting to grayscale to eliminate redundant color information, and downsizing to 128 × 128 pixels to reduce computational demand and memory footprint for the subsequent neural network training. The data is trained on a machine equipped with an Intel(R) Core(TM) i9-10900KF CPU with 3.70 GHz clock speed and an NVIDIA RTX 3080 Ti GPU. Network models are trained and tested on a Python environment using PyTorch with CUDA enabled.

### Network architecture and training

The neural network architecture we employ first passes the actuator input data through an LSTM network positioned between two deep feedforward networks (Figure 2). In an effort to further enhance noise resistance and improve interpolation properties, a generative model setup analogous to a Variational Autoencoder (VAE) is integrated into the model. Following the LSTM, the feedforward network splits into two networks, with their outputs connected to a Gaussian generative model. One network predicts the mean, and the other predicts the standard deviation for the sampler. The resulting sample is then directly rearranged into image format via reshaping.

The generated low-resolution image then passes through a tCNN decoder, which upscales it into the required grayscale 128 × 128 form. During predictions, the probabilistic model is turned off, with the mean feedforward layer output taken directly into the decoder. This modification ensures that control predictions from the model are completely deterministic, although retain the advantages of a VAE during training.

An MSE loss function was used with an *Adam* optimiser with a learning rate 10^–3^ (See Table 1). Normalisation and dropout were not used, and backpropagation through time (BPTT) was truncated to a maximum of 250 time points. Approximately 30 min were required to complete 2,500 epochs. The results demonstrate a potential for scalable implementation with larger datasets.

**TABLE 1:** Model training parameters.

Training epochs	6,000
Data sampling rate	30 Hz
Learning rate	0.001
BPTT truncation	300 time points
Final loss	0.01
Time for training	80 h
Number of training data points	4 batches of 36,156 time points
Number of validation data points	1 batch of 3,058 time points
Data points per sub-batch	150 time points
Final validation loss	0.0204

For the main training process, 4 temporal sequences comprising 36,000 data points each (36,156 data points after resampling) were gathered for the training dataset using motor babbling. A separate sequence of 3,000 data points (3,058 post-resampling) was collected for validation purposes. To concentrate the model’s training on the robot’s motion, the background was obstructed with a wooden board, thereby eliminating extraneous visual information. The model was then trained for a total of 6,000 epochs on the collected data, a process that took roughly 80 h to complete. Truncated BPTT used 300 frames of truncation while operating on data in sub-batches of 150 time points for lower memory requirements. The training parameters used in the preliminary testing for the final model were repeated for the final training.

### Controller design

In this work we employ a trajectory optimization algorithm for generating the control inputs. An optimization routine estimates the optimal input sequence $\hat{u_{i}}$ to achieve an output **Ψ**_*i*_ for the real system ( f,h ) using the forward dynamics model ( $\hat{\;f\;}$ , $\hat{h}$ ), estimated here using statistical machine learning approaches.

The controller calculates the optimal control inputs over a defined prediction horizon *T*, aiming to minimize a cost function. This cost function typically represents the discrepancy between the predicted outputs $\hat{\Psi }$ and the desired setpoints **Ψ**_des_, along with constraint penalties. In an open-loop setup, the control cycle is finished by applying the optimised inputs to the real system. The inclusion of a feedback term to close the loop would allow this process of optimisation to continuously repeat with time, adapting to changing conditions and disturbances.

Closed-loop feedback is dependent on the implementation of suitable hardware for shape capture, as well as a generally more complex controller design. An open-loop system is far simpler to implement, only requires one optimisation step, and is completely independent of the real system’s behaviour. However, the latter also implies that an open-loop controlled system has lower robustness to disturbances and model uncertainties.

Although operating on continuous systems, controllers are limited to discrete signals, constrained by the sampling rate between these and the system. The optimisation operates on a number of free input variables (maximum of *NT*, $u_{i}\in R^{N}$ for 0 ≤ *i* < *T*), attempting to find the input sequence that best matches the target. The target may be a shape at a specific time point (**Ψ**_des_) or a sequence of desired shapes at varying time points (**Ψ**_des,*j*_). At every optimisation step, a cost function is calculated that matches **Ψ**_des,*j*_ to the actual ${\hat{\Psi }}_{j}$ and return a scalar score. For the ideal cost function, a lower cost implies better shape matching.

In the context of images, the shape comparison involves identifying whether two images representing the robot’s configuration are similar. Although multiple techniques are available to measure image similarity, the images to be predicted are simple enough for mean squared error (MSE) to be reasonably appropriate in image comparison. To ensure that background effects are negligible, and the focus of the image comparison is on the robot’s shape, the background of the image is kept controlled as a constant throughout the data collection and testing. Eq. 1 defines the MSE cost between two images *I* and *J*:

$$C_{\text{MSE}}\left(I,J\right)=\underset{i}{\sum }\underset{j}{\sum }{\left(I_{ij}-J_{ij}\right)}^{2}$$ (1)

The optimiser’s role is to find an appropriate set of inputs such that $\sum_{j}C\left(\Psi_{\text{des},j},{\hat{\Psi }}_{j}\right)$ is minimised. However, high-dimensionality of image data, in addition to its non-linearity, may lead to numerous local minima in the overall cost function. A global optimiser avoids getting stuck at a local minimum by performing a wider search. Here we use *dual annealing*, or *generalised simulated annealing* (GSA), a powerful optimization algorithm inspired by the principles of thermodynamics, specifically the annealing process used in metallurgy (Xiang et al., 1997).

The most general optimisation for a shape change of *T* s with a 30 Hz sampling rate has 30*T* × 6 = 180*T* input variables to be optimised. This can quickly become very large, greatly increasing the time to optimisation. A more time-efficient alternative to this general model is to constrain the inputs to follow a piecewise linear form. In this setup, only a few *n* inputs at given time points are optimised by the controller, with the values of other inputs being determined via linear interpolation, or set constant at the end of the motion. Note that if *n* = 30*T*, then this approach reverts to the original general input form. More complex motions require a larger value of *n* to achieve better optimisation by allowing greater freedom to the input form.

Any control motion is started with the robot at its rest position and zero applied input. The predictions, however, show some transient behaviour at the beginning due to the presence of zero initial hidden states (state and memory). To cancel this effect, every control motion is first begun by appending 100-time steps of zero input prior to the start of control, allowing the transients to decay. A possible alternative to this method would be to run the model for a zero input and obtain the values of the hidden states following the decay of the transient, which can then be set as the new initial states. Since the tests to be performed are not dependent on high speed operations, the faster former solution is employed.

Although the background is constant, there is still the presence of noise due to lighting and model imperfections. To reduce the effect of these during optimisation, Otsu’s method for thresholding (Otsu, 1979) is applied to the images prior to comparing them with MSE (Figure 3). This automatic thresholding algorithm transforms the recorded images into binary data (black and white), removing the effect of small noise from the aforementioned sources in most of the pixel brightness range (except around the threshold point, which is a small region). Switching the VAE in the model during optimisation may also help in improving robustness to noise during control.

Although the long sequence LSTM network allows long-time effects such as hysteresis to play a role in the modelling, its effect is minimised in control by testing simple short motions spaced by a few minutes. This allows each motion to be tested against the model independently of previous tests.

## Results

### Training performance

The visuo-dynamic network is trained using four distinct video sequences, each spanning a duration of 20 min and recorded at a frequency of 30 Hz. A reduced validation dataset, around 2 min long, is employed to evaluate the effectiveness of the trained network. The loss function employed for both training and validation is the mean squared error (MSE) loss between the predictions generated by the model and the real image data. For context, instances where the average loss values between images remain below the threshold of 0.05 are indicative of satisfactory accuracy. Throughout the training phase, the average loss stabilizes at approximately 0.01, while the average validation loss converges around 0.02. Visual analysis indicates a tendency for the model’s predictions to exhibit higher errors in estimating the tip configuration. Consequently, shape errors primarily manifest in these specific regions, as demonstrated in Figures 2B, D.

**FIGURE 4:**
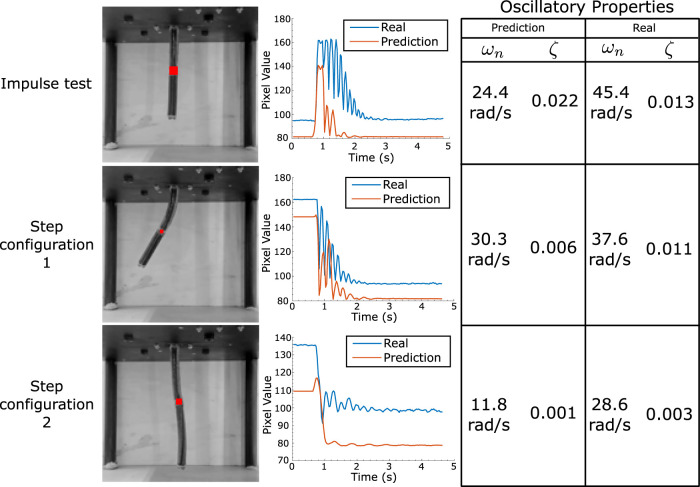
Oscillatory analysis of robot motion. The average pixel brightness at the robot’s half length point is measured for its final configuration after an impulse/step. This is plotted for both real and model motions, with the table on the right providing the estimate for both stiffness and damping factors at each configuration.

A more detailed investigation of the dynamical accuracy of the model is performed via a study of the oscillatory properties of both the model and the real robot. This examination centres on the determination of the relative stiffness and damping ratio across varied robot configurations. These values are estimated for two different step input configurations (see Figure 2D) by tracking the brightness changes of the image at a particular point in the robot near the final configuration. The calculated properties are given in Figure 2E. It can be seen that although the final predicted configurations are very similar, the learned model seems to have lower stiffness estimates, indicating possible model mismatch at higher frequencies. Given that the sampling rate for data acquisition is 188.4 rad/s (30 Hz), we expect by the sampling theorem to have all frequencies less than about 94.2 rad/s to be represented by the data. Since an RNN is always capable of representing the dynamic behaviour below this frequency (Schäfer et al., 2006), it is hypothesised that either data acquisition methods can be further refined to reflect on oscillatory information, or that more fine-tuned training is required to learn this information, which is left for further study. An in-depth description of this experiment is given in Figure 4, which shows the point at which the brightness change is measured and plots it with time, allowing for a graphical estimation of the oscillatory properties. Note that these tests have been performed in quick bursts, spaced by long time intervals as to reduce the effects of hysteresis on the motion.

### Shape tracking experiments

**FIGURE 5:**
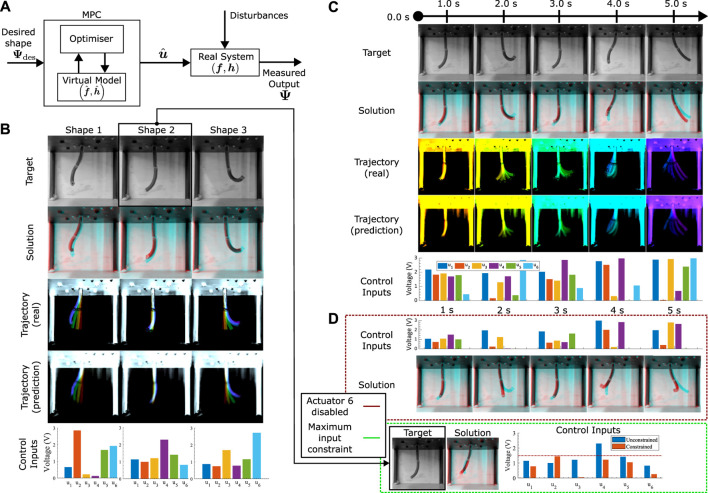
Control performance **(A)**. The optimization based control architecture used in this study. **(B)** Static control tests: A single final target is given to the optimiser, which chooses a 3-s ramp input to a set of 6 final actuator values that achieve an optimal match between the target and prediction at the last frame. The test is repeated three times, with the overall real and predicted trajectories plotted (time progression colourised with a jet colourmap, red to violet), in addition to the final 6 optimal actuator values. The final frame solution (red) is overlayed with the target (cyan), with matching pixels in black. **(C)** Dynamic control tests: Optimiser is given 5 shape targets at specific time points. The solution at each of the relevant time points (red) are overlayed with the targets (cyan), with matching pixels in black. Trajectories for the whole motion are illustrated (time progression colourised with a jet colourmap, red to violet). Optimal inputs are plotted for each of the target time points. **(D)** Generated motions with actuator constraints. Top: Dynamic targets from **(C)** with the 6th actuator disabled. Solutions (red) overlayed with the targets (cyan). Bottom: Static control with actuators limited to half of its maximum limit. Chosen target identical to Shape 2 in **(B)**. Comparison between free and constrained maximum inputs are plotted.

The control framework utilized in this study leverages the acquired visuo-dynamic model and an optimization algorithm to generate open-loop trajectories, as illustrated in Figure 5A. In this process, a global optimization algorithm is employed to determine the optimal actuator inputs over time, with the objective of minimizing a scalar quantity dependent on our control goals. This global optimization procedure utilizes a dual annealing approach (Xiang et al., 1997), with its core focus on minimizing the mean squared error (MSE) error function between the model-generated predictions and the target values. The targets are defined as sequences of desired shapes for the robot’s image at specific time points. In general, a qualitative assessment of the matching between images can be performed by observing the results directly. Nevertheless, such approach is not very scientific and we decide to use the mean squared error between target images and physical robot motion as a quantitative measure of tracking accuracy, as described in prior sections. To mitigate the influence of lighting and background noise, Otsu thresholding (Otsu, 1979) is applied to both the real images and the target images before computing the MSE.

The initial set of control assessments examines the controller’s capacity to achieve a fixed target shape during a 2-s control period (refer to Figure 5B). This evaluation places primary emphasis on achieving a static alignment of the ultimate shape, with the trajectory being of secondary significance. A visual examination of Figure 5B reveals a satisfactory alignment in the overall 2D shape, though there are slight deviations in the 3D positioning of the tip.

The next control test focuses on shape control within dynamic motions. The test illustrated in Figure 5C seeks to realize a sequence of target shapes spaced at 1-s intervals. The trajectory and motion planning now assumes a more important role, with the optimiser presented with the problem to manipulate input magnitudes at each designated time point (assuming a linear interpolation between these instances), to minimize a single scalar value that is a sum of the cumulative tracking error. The optimized inputs, illustrated at the bottom of Figure 5C, illustrate this modulated control strategy. Notably, the average MSE for all images remains below 0.25, indicative of good numerical accuracy. Visual comparison between the target and actual shapes (Figure 5C) reveals satisfactory alignment, albeit with persistent challenges in tip matching. It is important to note that one source of error in this experiment arises from the multi-objective optimization, where reducing the error for one target may lead to an increase in error for the other as the optimiser gets stuck in a local minimum, or the change between the dynamic shapes is infeasible and the optimiser minimises the MSE by worsening the shape match with some of the shapes. By using a global optimiser, we reduce the chances of such a scenario and increase the probability of an even target scaling in the error minimisation, although the infeasibility of the dynamic motion remains.

Moving forward, we delve into the analysis of control performance when additional constraints are introduced into the optimization problem. In Figure 5D, we visually represent these constraints, taking into account two distinct scenarios. The first scenario aims to replicate situations where the physical robot experiences damage to one or more actuators, resulting in one of the inputs remaining inactive. For the sake of brevity, we arbitrarily choose input 6 for the following tests. The goal here is to optimize shape matching while adhering to predefined constraints. Notably, as shown in Figure 5D (top), there is a noticeable improvement in aligning shapes that were previously less reliant on the 6th input. Knowledge of the STIFF-FLOP’s manufacturing shows that the 6th actuator has a large impact on the shape of the bottom module of the robot. This is clearly evident in Figure 5D, where shape errors primarily manifest at the tip. In the second scenario (illustrated in Figure 5D, bottom), the constraint necessitates the robot to achieve a static shape transformation to the “Shape 2” target within a 2-s timeframe, with each input limited to half of its maximum capacity. While an exact shape match is not achieved, the constrained outcome exhibits substantial alignment in orientation, accompanied by a comparably low mean squared error (MSE) as observed in the unconstrained test. The numerical values presented in Figure 5D indicate that the unconstrained values of inputs 2, 5, and 6 are restricted due to the imposed constraint, with input 6 being predominantly inactive. Actuators 1 and 3 experience minimal alterations, whereas actuator 4 undergoes a significant increase in voltage. This variation underscores the effectiveness of both the optimizer and the model in navigating the input space to identify alternative optima for this specific test scenario.

**FIGURE 6:**
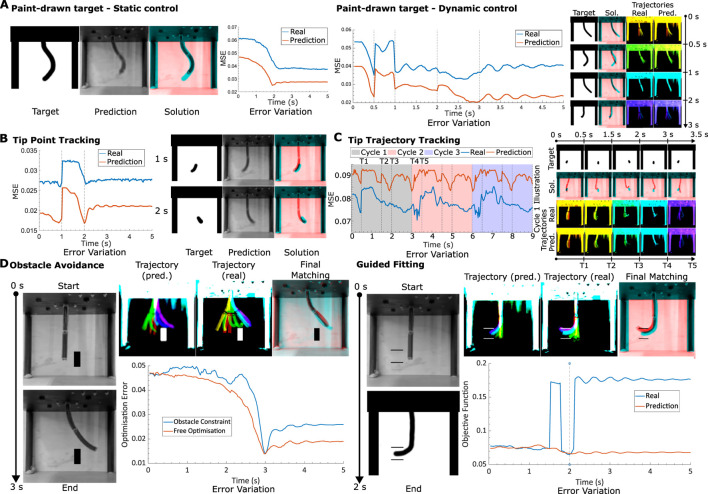
Method generalisability **(A)**. Hand-drawn targets. Left: A static test where the target image shape is to be reached in a time span of 3 s. Final solution (red) is overlayed with target (cyan). The MSE (relative to final target) variation with time is also plotted for both the model and real motions. Right: A dynamic test where 4 hand-drawn targets are to be reached in sequence within the same motion. Trajectory illustrated for both real and predicted motions, time progression colourised with a jet colourmap, red to violet. Final solution (red) is overlayed with target (cyan). MSE (relative to next target in time) variation with time for both model and real motions is also plotted. **(B)** Tip tracking. Left: Simple dynamic target, where tip is demanded to go from left to right. Solution and MSE (relative to next target in time) variation with time plotted. Right: Periodic dynamic motion. MSE (relative to next target in time) plotted for 3 cycles of the same targeted motion. Illustrations of the targets, as well as the solutions (target in cyan, solution in red) and trajectories (time progression colourised with jet colourmap from red to violet) for the first cycle, are given. **(D)** Obstacle control. Left: Static control with box obstacle in path. Final prediction and real trajectories illustrated (time progression colourised with jet colourmap from red to violet). Final matching, with cyan target and red solution also displayed. Variation in optimisation error is plotted for both the optimal inputs, with and without the constraint. Right: Precision static control, tip required to fit in between two vertically separated walls without collision. Final prediction and real trajectories illustrated (time progression colourised with jet colourmap from red to violet). Final matching, with cyan target and red solution also displayed. Variation in objective error (MSE with added obstacle penalties) is plotted for both real motion and model prediction. Steps in the objective error plot for real motion indicate collisions.

For all our previous tests, all the target robot shapes were sourced from the training or validation data. Expanding the scope of this investigation involves allowing the user to prescribe the desired robot shape, a notion particularly exemplified through hand-drawn renderings. By subjecting the robot to control targets composed of hand-drawn shapes, both static and dynamic control scenarios are tested (as illustrated in Figure 6A). Hand-drawn targets offer a heightened degree of shape diversity, yet the intrinsic challenge lies in matching such arbitrary shapes with the robot’s motion constraints. Hence, the controller’s aptitude for achieving feasible shapes that best match the designated targets becomes important.

**FIGURE 7:**
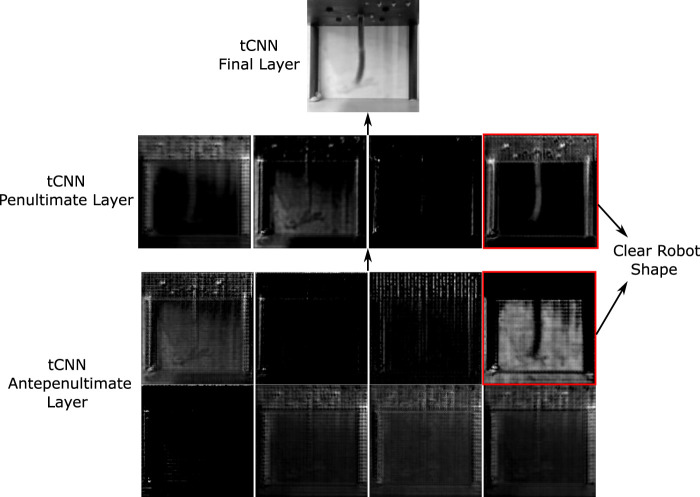
Display of intermediate transpose CNN layers. Last layer has one grayscale channel, obtained via the transpose convolution of the previous 4 channel layer. The layer before that (antepenultimate) has 8 channels. The robot’s shape is only clearly observable in only two of the channels in the previous layers, indicating a potential overdesign of the network for robot motion prediction.

Like before, the robot is first tasked with attaining a static target shape within a 2-s interval. Our results show reasonable alignment between the target and the achieved configuration, alongside a low static MSE. Note that now the mean squared errors are also affected by the background of the drawn image, which for certain cases can affect the desired user shape. The dynamic control domain unveils more intricate dynamics, exemplified by robust shape correspondence with targets at 0.5, 2, and 3-s marks, while demonstrating poorer alignment at the 1-s mark. It is evident that purely looking at the MSE values do not provide a good understanding about the tracking performance. The process occasionally leads to shortcuts taken by the optimizer to prioritize pixelwise alignment at the expense of shape fidelity—a behaviour expected from our simple loss function, that warrants refinement for better performance. A method to ignore the background of the image is also required for a smoother, consistent loss curve. Potential solutions for this could involve utilizing the model’s latent space representation, which automatically achieves this (Figure 7).

Furthermore, the incorporation of hand-drawn targets introduces possibilities for more conventional control tasks like tip tracking. In the next test, we modify the target to focus on tip tracking, permitting the controller to adapt the rest of the robot’s configuration to optimally align with the desired tip target. Figure 6B illustrates outcomes for both a straightforward dynamic tip motion and a more intricate tip-tracking trajectory. The former demands the tip to traverse from right to left within 1 s, yielding satisfactory shape matching. Notably, the hand-drawn targets lack direct constraints on the robot’s 3D motion, thus allowing the target to be achieved in diverse 3D orientations. It is noteworthy that for the 2-s target, the solution orients the robot backwards—an illustrative demonstration of this flexibility. The observation further reveals that while a minimum in MSE is achieved at the required 2-s mark, subsequent deviations occur along with oscillatory behaviour as the robot converges toward its steady state. This specific observation demonstrates the controller’s capacity to exploit transient dynamics to minimise errors.

We now extend our tip tracking experiments to cyclic motions, where the robot’s tip sways from left to right, over multiple cycles (Figure 6C). The MSE variation over time aligns mostly with the periodic nature of each cycle (period of 3 s), indicating near-perfect periodicity in predictions without decay over time. We also observe a limit-cycle-like convergence of the motions within one cycle for both the learned and real systems.

The conceptual foundation laid by the hand-drawn control approach readily accommodates the establishment of a system for generalized obstacle avoidance. In the context of robot control, obstacle avoidance is pivotal for trajectory planning and can be extrapolated to a range of higher-level constraints in generalized systems. One test involved directing the robot to navigate around a hand-drawn box object while following a trajectory toward a final shape. Any overlap between the robot’s black pixels [determined through Otsu’s thresholding (Otsu, 1979)] and the obstacle area results in a substantial MSE cost penalty. As a consequence, the optimizer must chart a trajectory that avoids the obstacle. The outcomes of this evaluation are depicted in the left portion of Figure 6D. It is discernible that the robot adeptly employs its full range of motion capabilities to circumvent the obstacle, even though the predictions are all in 2D. The optimization error plot highlights how the error between the current and desired shapes evolves with time, revealing that the imposition of constraints leads to trajectories with higher error compared to unconstrained, constraint-free trajectories.

The next obstacle avoidance test challenges the robot to align its tip between two vertically spaced walls, resembling a cross-sectional view of a cylinder, for instance (see Figure 6D, right). The modeled predictions exhibit commendable behavior; however, in the real-world test, collisions with the non-existent walls become evident. Similar to the initial test, collisions incur a cost penalty, resulting in spikes within the objective function value for the real scenario—observable in the plot in Figure 6D, right. The model’s intrinsic uncertainties regarding tip behavior emerge as a significant source of error in precision fitting tasks, with underestimations in tip length and position contributing to inaccurate collision predictions.

Throughout Figure 6, the prediction data has a lower MSE than the real data and hence predicts better control. The target shapes for these curves are all given by real robot shapes, and hence are prone to variations in local brightness. The MSE with the model, which is an average with the training data, will therefore be non-zero at the same robot shape due to these local pixel variations. Nevertheless, the shapes of the curves are very similar, suggesting that the overall target shapes are indeed being tracked. Better metrics than the MSE would improve the current method by better normalising the trained images through more complex filtering.

### Model accuracy experiments

**FIGURE 8:**
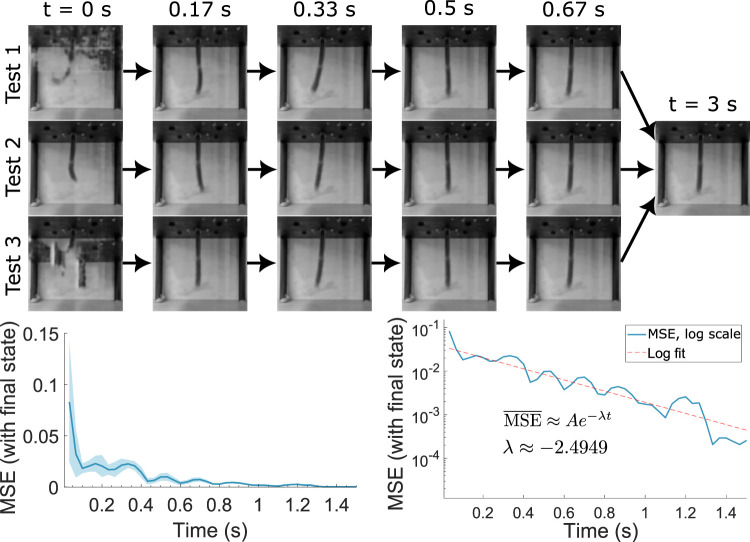
Steady state decay from random initial LSTM state. Bottom left plots the MSE relative to the final state with time averaged for multiple tests, together with the maximum/minimum bounds. Bottom right plots the average on logarithmic scale, allowing for an estimation of the decay parameter.

The model uses a long short-term memory (LSTM) model to represent the dynamics of the system. Such models are characterised by their ability to retain necessary information for long periods of time, while “forgetting” other aspects that are less important for the motion. Although normal recurrent neural networks are capable of replicating this behaviour, the specific inclusion of a memory gates in the LSTM architecture has been demonstrated to improve their training properties. Nevertheless, we can use the memory property of the system to test its decay to steady state, as given in Figure 8. Here we initialise the steady state of the network with a random value for many iterations at zero input, and test the time to decay to the stationary initial state. The MSE with respect to the stationary state results are plotted in the graphs at the bottom of Figure 8, which suggest an average decay rate of around −2.49 s^−1^. This result indicates that in the motion data provided, current time points are dependent on about 1.84 s of data prior to the time point on average, which is the time taken for a 99% MSE drop. This provides insight on the real robot’s memory properties and allows us to further optimise computational resources when using backpropagation through time (BPTT) in the model to match the system’s requirements and properties.

**FIGURE 9:**
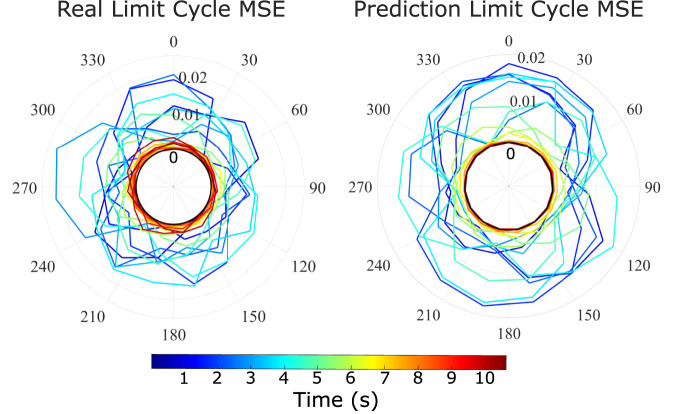
Limit cycle stabilisation in real robot and prediction model. Model and real robot are subjected to two limit cycles with initial noise. The relative MSE between the two motions between model and model, real and real, are plotted in the radial plots above. One cycle of the radial plot equals to 0.5 s.

One important property of non-linear systems such as the tested soft robot are the existence of limit cycles. We test this property on our model and compare it to the real robot by providing the system with oscillatory inputs, superposed with Gaussian noise (identical for both real and model predictions) in the first few seconds of the motion. Figure 9 demonstrates the results for the real system and the prediction, plotting the MSE to the noiseless cyclic motions. Each cycle is given by a single 360° of the plot. We note that both plots tend to zero MSE after the noise is removed at around 5 s of the motion, which is expected from the decay property of the system. We clearly observe some difference between the real and prediction limit cycles during the noise action, with the prediction cycle being much more symmetric. This can be attributed to the averaging action of the model and included variational auto-encoder setup, which leads to smoother behaviour in the action of noise disturbances. Both model and real data have a maximum MSE deviation of around 0.02 from the final steady state cycle, and seem to decay at the same rate, reaching this steady state after around 7 s. These agreements show good predictive abilities in limit cycle behaviour.

It is also instructive to understand how the model predicts the shape change from the actuator inputs directly. Neural networks are functional approximators and can give insights into the important features of the system that can be hard to directly model under constrained motion. Figure 3 displays the outputs of the final transpose convolutional network (tcNN) layers that compose the model’s decoder. We note that most of the layers in the trained model are used to solely predict the background changes, while the shape information is coded into 1 or 2 channels. This informs us that the model can be greatly simplified for further computational optimisation via the removal of unnecessary background information. Furthermore, the transpose convolutional kernels used in the channels containing the soft robot information can provide insight into the key aspects of the function approximation, which is left for potential future work.

## Discussion

In this research, we introduce an end-to-end learning-based approach that enables the comprehensive dynamic modeling of a broad spectrum of robotic systems. The core concept revolves around acquiring dynamic models of the robot directly within the visual domain. To validate the effectiveness of our proposed method, we apply it to a fully soft robotic manipulator. We showcase its practicality in controller development through an open-loop optimization-based controller. Our approach successfully accomplishes dynamic shape control, trajectory tracking, and obstacle avoidance using a model derived from just 90 min of real-world experience.

In the field of soft robotics, the approach we present is, to the best of our knowledge, the first instance of a learned controller for shape control. Unlike model-based techniques, our method does not require any prior knowledge about the system. Furthermore, unlike other model-free approaches, our framework only requires a simple setup, consisting of an inexpensive RGB camera and the robotic system itself. Control targets can be specified by users with minimal knowledge about the robot’s kinematics and without the need for a global coordinate system. Additionally, the framework allows users to draw target points and shapes, making it highly accessible to non-experts and potentially applicable in the field of robotic surgery and inspection in tight spaces.

Due to the novelty of the proposed approach, there are no suitable comparisons to previous works that can be done to evaluate our model’s effectiveness against literature. Its effectiveness is shown through a qualitative view and an analysis of the mean squared error between the real system and prediction, which is again arbitrary. Further works are encouraged to improve our approach and introduce better metrics for control effectiveness.

While the presented model is tested on a single robotic system, the method is applicable to any dynamical system as long as they do not exhibit highly chaotic behavior. A future extension of this work would involve learning models for stereo images and multi-modal dynamic models, such as predicting visual and contact dynamics. One of the challenges in this setting would be representing targets for the controller. A possible solution could involve using the learned model itself to generate target images conditioned on the VAE parameters. Currently, our results are validated using an open-loop controller due to the time cost of the optimization routine. Reducing this computational time is a future research direction.

## Data Availability

The code and trained model used in this study are publicly available. This data can be found here: https://github.com/RichieRich2569/4YP-Dynamic-LSTM.
